# Local scientific relevance in the face of the tyranny of global metrics: the future of the diamond model and health evidence needs in the region

**DOI:** 10.17843/rpmesp.2026.432.16722

**Published:** 2026-06-30

**Authors:** Mauricio Palacios Gómez

**Affiliations:** 1 Associate Editor of the journal Colombia Medica.; 2 Universidad del Valle, Cali, Colombia.

In recent decades, the editorial systems for evaluating science in Latin America (LA) have undergone a transformation by adopting international standards that prioritize international bibliometric metrics and indexes such as Web of Science and Scopus ^1^. The historical evolution of Publindex in Colombia clearly illustrates this transition: this index was initially conceived to register and make visible local scientific production; subsequently, it was integrated into academic incentive systems, which promoted the increase in university journals. Given the increase in the number of publications, the Colombian State opted to add a regulatory function to Publindex, which implied the adoption of external standards from the bibliometric indexes of Web of Science and Scopus ^2^.

The adoption of global standards without adequate contextualization for LA modified publication practices and editorial decisions, which, although it accelerated the evaluation process, also conditioned local journals to focus primarily on obtaining citations and optimizing indicators such as the impact factor or the H-index. Thus, the simplicity and dependence on a single indicator (the bibliographic citation) suggest that these metrics have become objectives and reflect Goodhart's Law, according to which, «when a measure becomes a target, it ceases to be a good measure » ^3^^,^^4^. Citation-based metrics, such as the number of citations, the H-index, or the impact factor, have ceased to be a good indicator because they are biased by the volume of articles, self-citations, and the increase in bibliographic references. Currently, these metrics are not useful for comparing researchers from different fields or within the same department ^3^.

In this model, local journals assume high institutional costs to meet increasingly strict indexing requirements and compete under unequal conditions against journals from the Northern Hemisphere with greater financial resources, many of which belong to the same publishing group that develops said metrics and bibliometric indices ^5^. Although state funding for local scientific journals could help reduce these technical costs, the budgetary limitations of LA countries restrict this option. This reality forces researchers to resort to commercial open access models through the payment of article processing charges (Article Processing Charge, APC). For example, Colombian researchers allocated USD 11.98 million to APC payments, with an average of USD 1,593 per article between 2013 and 2020, which reduces the budget for science ^5^.

In the region, the concern is similar. The phrase «publish or perish… and pay » summarizes the concerns regarding the APC issue in Brazil ^6^. The authors point out that the current commercial open access model forces researchers to allocate resources originally intended for knowledge production to pay publication costs.

This dependence on global metrics is aggravated by the lack of regulation of APC or explicit criteria for financing these charges. As a consequence, the national science system faces an economic «triple burden»: financing research, sustaining costly subscriptions to scientific journals or commercial publishers, and assuming the costs of open access publication ^7^. An even more problematic scenario could arise in which some institutions choose to allocate resources to the payment of APC to increase their visible output and institutional indicators. This strategy could incentivize publication in journals of questionable editorial quality or with predatory practices. In economic terms, the cost of financing the publication of an article through APC is usually much lower than that of developing a complete research project and involves fewer risks. If evaluation systems prioritize the number of publications over the quality or impact of research, incentives could be generated to preferentially invest in the publication of results rather than in the production of new knowledge.

In the field of health, this dynamic deepens inequalities in the generation of scientific evidence. By conditioning publication on the ability to pay or alignment with the interests of large publishing groups in the Northern Hemisphere, this dynamic favors that commercial priorities do not necessarily coincide with the epidemiological needs of Latin American countries. Added to this is the low valuation of regional research when it is published in journals that the system itself recognizes as having lower prestige in metrics-based classification systems. As a consequence, many diagnostic and therapeutic guidelines are adopted based on evidence generated in other regions, without sufficiently incorporating the epidemiological, social, and health findings documented by researchers in the region.

In the face of the crisis of the APC payment commercial model, there are regional strengths that could favor the development of less imperfect models for measuring scientific output. In Latin America, the diamond publishing model predominates (neither authors nor readers incur costs for publishing or accessing content), which has allowed initiatives such as SciELO, RedALyC, and Latindex to consolidate and grow by promoting editorial quality standards and regional visibility in line with the region's needs ^2^. The advantages of the diamond model are numerous. This model preserves the institutional autonomy of journals, especially those publications that fulfill clinical, teaching, scientific, and public health functions, whose social and academic value is not always reflected in high international citation indexes. This alternative is very difficult to develop in Northern Hemisphere countries, where editorial production is concentrated in a few publishing groups. Therefore, the region has the historical opportunity to lead and defend the development of alternative measurement models before APC-based systems become widespread and destroy the public scientific communication ecosystem.

From that perspective, the single-criterion approach to bibliographic citation must be replaced by a comprehensive multi-criterion evaluation. A relevant scientific evaluation proposal adapted to the needs of regional health would have at least three components. The first would correspond to international circulation, combining presence in PubMed and PubMed Central, as well as inclusion in the Web of Science and Scopus citation indexes. However, it carries the risk of turning indexing in PubMed Central or visibility in PubMed into goals in themselves (Goodhart's law again) ^8^. The second component should reflect the regional value of journals, based on indicators such as regional citation, language, relevance to local or regional health problems, and the adoption of the diamond open access model.

The third component would be related to editorial efficiency and would evaluate the classic criteria of editorial quality, with the addition of indicators of capacity to disseminate knowledge, promote its utilization, and optimize editorial processes that reduce barriers or facilitate increasingly complex editorial processes. The guideline consists of attracting regional production, avoiding the shelving of research, and promoting the use of scientific production. There could be more indicators, and it might possibly be necessary that the construction of these indicators be independent among the areas of knowledge so that it better represents the ways of producing and communicating science.

Latin America has the human, academic, and editorial capacities necessary to build alternatives to evaluation models predominantly based on metrics that allow for a comprehensive and qualitative assessment of biomedical production. To do this, it is necessary to recognize and value the work of our scientists and editors, take advantage of the opportunity offered by the predominance of the diamond model before APC-based systems become widespread in the region, and reframe a fundamental question: why do we publish science? The answer should not be to accumulate citations or to advance in ranking systems. We publish to generate useful knowledge, guide health policies, improve professional training, strengthen clinical practice, and contribute to the well-being of our populations. We also publish because research generates social and economic returns that far exceed the costs of its funding. Recovering this perspective would allow for the construction of evaluation systems more aligned with the needs of the region and with the true purpose of scientific activity.
