# Supplementary material for: Local scientific relevance in the face of the tyranny of global metrics: the future of the diamond model and health evidence needs in the region
Source: Rev Peru Med Exp Salud Publica. 2026 Jun 30;43(2):158–9. doi: 10.17843/rpmesp.2026.432.16722 (PMC13456918; doi:10.17843/rpmesp.2026.432.16722)
Supplement: Translation

# Translation: Pertinencia científica local frente a la tiranía de las métricas globales: el futuro del modelo diamante y de las necesidades de evidencia en salud en la región

**Authors:** Mauricio Palacios Gómez

**Affiliations:** 1 Editor asociado de la revista Colombia Médica.; 2 Universidad del Valle, Cali, Colombia.

En las últimas décadas, los sistemas editoriales de evaluación de la ciencia en América Latina (AL) han experimentado una transformación al adoptar estándares internacionales que privilegian las métricas e índices bibliométricos internacionales como Web of Science y Scopus ^1^. La evolución histórica de Publindex en Colombia ilustra con claridad esta transición: este índice fue concebido inicialmente para registrar y visibilizar la producción científica local, posteriormente, se integró a los sistemas de estímulo académico, lo que impulsó el incremento de revistas universitarias. Ante el aumento del número de publicaciones, el Estado colombiano optó por añadir una función reguladora a Publindex, lo que implicó la adopción de estándares externos de los índices bibliométricos de Web of Science y Scopus ^2^.

La adopción de estándares globales sin una adecuada contextualización para AL modificó las prácticas de publicación y las decisiones editoriales, lo que, si bien aceleró el proceso de evaluación, también condicionó a las revistas locales a orientarse principalmente hacia la obtención de citas y la optimización de indicadores como el factor de impacto o el índice H. Así, la simplicidad y dependencia de un único indicador (la cita bibliográfica) sugieren que estas métricas se han convertido en objetivos y reflejan la Ley de Goodhart, según la cual, «cuando una medida se convierte en un objetivo, deja de ser una buena medida» ^3^^,^^4^. Las métricas basadas en citas, como el número de citas, el índice H o el factor de impacto, han dejado de ser un buen indicador porque están sesgadas por el volumen de artículos, las autocitas y el aumento de referencias bibliográficas. Actualmente, estas métricas no son útiles para comparar investigadores de diferentes campos ni dentro de un mismo departamento ^3^.

En este modelo, las revistas locales asumen elevados costos institucionales para cumplir con las exigencias de indexación cada vez más estrictas y compiten en condiciones desiguales frente a revistas del hemisferio norte con mayores recursos financieros, muchas de las cuales pertenecen al mismo grupo editorial que desarrolla dichas métricas e índices bibliométricos ^5^. Si bien la financiación estatal de las revistas científicas locales podría contribuir a reducir estos costos técnicos, las limitaciones presupuestales de los países de AL restringen esta opción. Esta realidad obliga a los investigadores a recurrir a los modelos de acceso abierto comercial a través del pago de cargo por procesamiento de artículos (Article Processing Charge, APC). Por ejemplo, los investigadores colombianos destinaron USD 11,98 millones al pago de APC, con un promedio de USD 1,593 por artículo entre 2013 y 2020, lo que reduce el presupuesto para ciencia ^5^.

En la región, la preocupación es similar. La expresión «publica o perece… y paga» resume las preocupaciones sobre la problemática de los APC en Brasil ^6^. Los autores señalan que el modelo actual de acceso abierto comercial obliga a los investigadores a destinar recursos originalmente destinados a la producción de conocimiento al pago de los costos de publicación.

Esta dependencia de las métricas globales se ve agravada por la falta de regulación de los APC o de criterios explícitos para financiar estos cargos. Como consecuencia, el sistema nacional de ciencia enfrenta una «triple carga» económica: financiar la investigación, sostener las costosas suscripciones a revistas científicas o editoriales comerciales y asumir los costos de publicación en acceso abierto ^7^. Incluso podría darse un escenario aún más problemático en el que algunas instituciones opten por destinar recursos al pago de APC para incrementar su producción visible y sus indicadores institucionales. Esta estrategia podría incentivar la publicación en revistas de calidad editorial cuestionable o con prácticas depredadoras. En términos económicos, el costo de financiar la publicación de un artículo mediante APC suele ser muy inferior al de desarrollar un proyecto de investigación completo y conlleva menos riesgos. Si los sistemas de evaluación privilegian el número de publicaciones por encima de la calidad o el impacto de la investigación, podrían generarse incentivos para invertir preferentemente en la publicación de resultados antes que en la producción de nuevo conocimiento.

Para el área de la salud, esta dinámica profundiza las desigualdades en la generación de evidencia científica. Al condicionar la publicación a la capacidad de pago o a la alineación con los intereses de los grandes grupos editoriales del hemisferio norte, esta dinámica favorece que las prioridades comerciales no coincidan necesariamente con las necesidades epidemiológicas de los países latinoamericanos. A ello se suma la escasa valoración de la investigación regional cuando se publica en revistas que el propio sistema reconoce como de menor prestigio en los sistemas de clasificación basados en métricas. Como consecuencia, muchas guías diagnósticas y terapéuticas se adoptan a partir de evidencia generada en otras regiones, sin incorporar de manera suficiente los hallazgos epidemiológicos, sociales y sanitarios documentados por los investigadores de la región.

Frente a la crisis del modelo comercial de pago de APC, existen fortalezas regionales que podrían favorecer el desarrollo de modelos de medición de la producción científica menos imperfectos. En América Latina predomina el modelo de publicación diamante (ni los autores ni los lectores asumen costos por publicar ni por acceder a los contenidos), que ha permitido que iniciativas como SciELO, RedALyC y Latindex se hayan consolidado y crecido, al promover estándares de calidad editorial y visibilidad regional acordes con las necesidades de la región ^2^. Las ventajas del modelo diamante son múltiples. Este modelo preserva la autonomía institucional de las revistas, especialmente de aquellas publicaciones que cumplen funciones clínicas, docentes, científicas y de salud pública, cuyo valor social y académico no siempre se refleja en altos índices de citación internacional. Esta alternativa es muy difícil de desarrollar en los países del hemisferio norte, donde la producción editorial se concentra en unos pocos grupos editoriales. Por lo tanto, la región tiene la oportunidad histórica de liderar y defender el desarrollo de modelos alternativos de medición antes de que los sistemas basados en APC se generalicen y destruyan el ecosistema público de comunicación científica.

Desde esa perspectiva, el enfoque monocriterio de la cita bibliográfica debe sustituirse por una evaluación multicriterio integral. Una propuesta de evaluación científica pertinente y adaptada a las necesidades de la salud regional tendría al menos tres componentes. El primero correspondería a la circulación internacional, combinando la presencia en PubMed y PubMed Central, así como la inclusión en los índices de citas Web of Science y Scopus. Sin embargo, conlleva el riesgo de convertir la indexación en PubMed Central o la visibilidad en PubMed en objetivos en sí mismos (otra vez la ley de Goodhart) ^8^. El segundo componente debería reflejar el valor regional de las revistas, basado en indicadores como la citación regional, el idioma, la pertinencia para los problemas de salud locales o regionales, y la adopción del modelo de acceso abierto diamante.

El tercer componente estaría relacionado con la eficiencia editorial y evaluaría los criterios clásicos de calidad editorial, con la adición de indicadores de capacidad para divulgar el conocimiento, promover su utilización y optimizar los procesos editoriales que reduzcan las barreras o faciliten los procesos editoriales cada vez más complejos. La directriz consiste en atraer la producción regional, evitar el engavetamiento de las investigaciones y fomentar el uso de la producción científica. Podrían ser más indicadores, y posiblemente sea necesario que la construcción de esos indicadores sea independiente entre las áreas de conocimiento para que represente mejor las formas de producir y comunicar la ciencia.

América Latina dispone de las capacidades humanas, académicas y editoriales necesarias para construir alternativas a los modelos de evaluación basados predominantemente en métricas que permitan valorar la producción biomédica de forma integral y cualitativa. Para ello, es necesario reconocer y valorar el trabajo de nuestros científicos y editores, aprovechar la oportunidad que ofrece el predominio del modelo diamante antes de que los sistemas basados en APC se generalicen en la región, y replantear una pregunta fundamental: ¿para qué publicamos ciencia? La respuesta no debería ser acumular citas ni para ascender en los sistemas de clasificación. Publicamos para generar conocimiento útil, orientar las políticas de salud, mejorar la formación de los profesionales, fortalecer la práctica clínica y contribuir al bienestar de nuestras poblaciones. También publicamos porque la investigación genera retornos sociales y económicos que superan ampliamente los costos de su financiación. Recuperar esta perspectiva permitiría construir sistemas de evaluación más alineados con las necesidades de la región y con el verdadero propósito de la actividad científica.
